# Supplementary material for: Are carbon emissions trading and green financial instruments synergistic? -Comprehensive quantitative research based on content analysis
Source: PLoS One. 2024 Mar 7;19(3):e0298601. doi: 10.1371/journal.pone.0298601 (PMC10919601; doi:10.1371/journal.pone.0298601)
Supplement: S1 Data — (ZIP) [file pone.0298601.s001.zip › Data/地级市Z_F工作报告.zip!/░▓╗╒/╨√│╟╩╨/└┤╫╘╣1⁄2╓┌║┼í╛┬φ┐╦╩2╛▌═°í┐_2004.docx]

——2004年2月25日在宣城市第一届人民代表大会第五次会议上

市长 高登榜

各位代表：

现在，我代表市人民政府向大会作政府工作报告，请予审议，并请市政协委员和其他列席会议的同志提出意见。

过去的2003年，在省委省政府和市委的领导下，全市人民坚持以邓小平理论和“三个代表”重要思想为指导，认真贯彻落实党的十六大和十六届三中全会精神，聚精会神搞建设，一心一意谋发展，克服了非典疫情和局部旱灾的不利影响，整体经济呈现出增速加快、质量提高、亮点增多的良好发展态势，各项社会事业同步推进。

经济持续快速增长。

全年完成国内生产总值205.3亿元，比上年增长9.3%。财政总收入12.37亿元，增长12.5%，其中地方财政收入7.33亿元，按可比口径增长15.3%。农业和农村经济稳步发展，全年粮食总产96.３万吨，油菜籽总产13万吨，棉花、烟叶、蔬菜、花卉苗木、中药材等经济作物面积进一步扩大，畜牧水产业和经济林果业发展继续加快，退耕还林任务超额完成。农业产业化经营保持全省领先水平，宁国市被列为省农业产业化示范县，广德被列为省新型农民专业合作组织试点县，全市有14家企业进入省级农业产业化龙头企业行列，广德和威集团被列为国家级农业产业化龙头企业。农业标准化生产有了新的进步，我市被列为全国农产品标准化综合示范区，83个品种获得省级名牌农产品和无公害、绿色、有机食品标志认证，比上年增加46个。工业经济增速加快，质量和效益不断提高。规模以上工业增加值增长16.8%，全社会用电量增长40.8%，工业用电量增长82.07%，工业经济效益综合指数达到136.7，比上年提高27.6个百分点。企业技术改造和技术创新步伐加快，全市共实施重点技术改造项目20项，实施省以上技术创新项目32项，开发新产品228个。旅游业在非典消退后迅速恢复，房地产业保持快速增长，商贸流通、交通运输、邮政通信等行业稳步发展。全年社会消费品零售总额66.23亿元,比上年增长6.8%。金融保险业稳健运行，年末金融机构存贷款余额分别比年初增加26.18亿元和12.99亿元。非公有制经济发展加快，全市个体工商户达到6.02万户，私营企业发展到4341家,个体私营经济占全市经济总量的份额达到40%左右。

改革开放加速推进。

列入省政府考核的60户国有集体企业已有52户完成改制任务。市直企业改革强力推进，驻芜企业职工安置工作基本完成，资产盘活工作正在全面展开。农村税费改革规范年活动扎实推进，农业特产税改征农业税，直接减轻农民负担2216.7万元；粮食补贴方式改革试点全面推开，向农民直接发放补贴款1663万元。市县公共财政支出改革进一步深化和完善。市直卫生事业单位机构及人事制度改革和宁国市综合执法改革被列入省试点。乡镇行政区划调整工作全面完成，全市农村乡镇由164个调整为100个，撤并比例达39％。宁国、郎溪、广德、泾县、绩溪已完成撤并村工作。住房公积金管理机构调整、人员分流和资产移交工作全面完成。招商引资成效显著，全市共新批外商投资项目24个，28个项目有外资到位，实际利用外资3815万美元，比上年增长60%；引进省外投资项目845个，实际到位省外资金28.9亿元，增幅居全省第一位，总量居全省第三位。对外贸易保持快速增长，全市外贸进出口总额达到1.24亿美元，比上年增长46.2%，其中出口8727万美元，增长42.1%。经济技术合作与交流不断扩大，我市与江苏南京市、上海闵行区先后建立了友好市区关系。

固定资产投资增势强劲。

争取国债资金成效显著，新开工项目不断增多。全年完成固定资产投资60.4亿元，比上年增长81.1%。水利基础设施建设步伐加快。马山埠闸、双桥闸建成将投入运行。交通“九路八桥”工程加速推进，芜宣高速公路宣城段、205国道泾南段、104省道河唐线改造、322省道宣泾线琴溪复桥、幕山复桥等已建成通车，318国道广祠段高速公路、宣杭铁路复线、214省道望十线、215省道广宁线及宁国排湾店至绩溪38号桥段、230省道广溧线改造和县乡公路“通达工程”等一批重点项目正在抓紧建设之中。城市规划、建设和管理工作进一步加强，《宣城市城市总体规划》修编工作已完成，城市近期建设规划成果已通过评审，红星礼堂、市人民医院新病房楼竣工并投入使用，梅溪路改造、宣港路出口段、皖东南救灾物资储备中心、市农科所新址建设主体工程、市消防指挥中心、宣城日报社综合楼等项目基本建成。昭亭南路、环城大道西南段、敬亭山东大门改造等重点市政工程项目进展顺利。宣城经济技术开发区建设取得重大进展，基础设施建设全面推进，功能性建设加快实施，城市新区框架逐步拉开。城市经营性用地招标、拍卖和挂牌出让工作取得新的进展，全市土地收益达到5.7亿元。市区交通秩序综合整治工作力度加大，取缔二轮“摩的”运营，一举解决了多年困扰市区交通管理的老大难问题，市容市貌有了新的改观。按照徽派建筑风格建设农村住宅工作启动实施。

城乡居民生活水平继续改善。

全市城镇居民人均可支配收入6457元，比上年增长11.7%。农民人均纯收入2502元，增长4.3%。城乡居民储蓄存款余额达103.13亿元，比年初增加16.8亿元。就业和再就业工作稳步推进，市场化就业机制基本形成。全年新增就业岗位18389个，安置下岗失业人员再就业12106人，其中“4050”人员实现再就业1322人，城镇登记失业率控制在4%以内。国有企业下岗职工基本生活费和离退休人员养老金做到按时足额发放，基本养老、失业、医疗等社会保险覆盖面继续扩大，全市企业退休人员社会化管理率达到84%。城乡居民最低生活保障制度得到加强，全市分别有35464人和16430人被列入城镇和农村最低生活保障对象，累计发放最低生活保障金2702.4万元。扶贫开发工作取得新的进展，全年争取扶贫资金6945万元，扶持项目158个，贫困地区的群众生活逐步改善。农村劳动力转移步伐加快，全年农村劳务输出近40万人，劳务收入占农民人均纯收入的比重达到21.5％。

各项社会事业和民主法制建设取得新的进展。

科技事业发展加快。完成省以上重大科技成果20项，新批认定省级高新技术企业2家、省级民营科技企业16家，宁国市科技进步综合考核位居全省第一，农业科技成果转化“1515”工程有效实施。教育事业发展势头良好，基础教育得到加强，素质教育全面推开,职业教育保持全省领先水平。中小学布局调整和危房改造工作成效显著,全年完成中小学D类危房改造22.1万平方米。农村义务教育管理体制、办学体制和基础教育新课程改革稳步实施。人口与计划生育综合改革继续向纵深推进，人口出生率和自然增长率分别为8.3‰和2.91‰，保持了全省先进水平。艺术创作和群众性文化活动更加丰富，文化市场管理和文物保护工作进一步加强。广播电视覆盖面继续扩大，传输质量和节目质量不断提高。全民健身运动广泛开展，成功举办了市（区）直机关首届运动会。卫生事业健康发展，新型农村合作医疗制度试点工作稳步推进。重视国土资源管理和环境保护工作，集中整治河道开采砂石，河滩风景资源开始得到保护。“一控双达标”成果继续巩固，省下达的4项主要污染物年度排放总量控制目标基本实现。“生态宣城”建设正式启动，全市已有5个县市区被列为国家级生态示范区试点，南漪湖流域环境污染综合整治和生态保护规划编制工作全面展开。社区建设得到加强，区街经济有了新的发展。公民道德教育和精神文明创建活动逐步深入，“四五”法制宣传教育有效开展。整治和改善经济发展环境工作继续向纵深推进，市、县两级政务服务中心网络基本形成。广泛开展政风评议和创建“人民满意的基层站所”活动，政府部门的行政行为进一步规范。市政府全年共办理人大代表议案1件、建议53件，政协委员提案66件,办理质量和效率不断提高。社会治安综合治理力度加大,人民来信来访工作进一步改进，群体性事件和集体上访得到及时稳妥处理。注重从源头预防和治理腐败，公款吃喝现象有所遏制,“双清”工作取得显著成效。国防后备力量建设和“双拥”工作得到加强，成功举行了第一次平战转换演练。审计、统计、物价、气象、地震、民族、宗教、外事、侨务、档案等各项工作都取得了新的成绩。

面对突如其来的非典疫情，各级政府坚持把人民群众的身体健康和生命安全放在第一位，按照党中央、国务院和省委省政府的统一部署，及时建立了覆盖城乡的疫情监测报告体系和诊断救治体系，构筑全民动员、群防群治的防治网络，卓有成效地组织开展抗击非典斗争，取得了“非典病人”和“疑似病人”零发生的重大阶段性胜利。非典过后，根据全国防治非典工作会议精神，对去冬今春的非典防治工作及早进行了研究和部署，进一步加强公共卫生体系建设，抓好经常性防治措施的落实，公共卫生突发事件应急处理和防控长效机制初步建立。

各位代表，过去的一年，我们坚持把融入苏浙沪经济圈作为经济发展的主战略，不断加强同苏浙沪地区的横向联系与协作，主动接受苏浙沪的经济辐射，使融入战略逐步得到深化；坚持把招商引资作为经济工作的首位任务，严格落实目标责任制，多渠道、多形式开展招商引资活动，促进了经济的即期增长和长远发展；坚持以加速工业化为核心，统筹城乡发展，大力推进经济结构战略性调整，加快产业升级和总量扩张，工业经济的主体地位更加突出；坚持把发展民营经济作为推动县域经济发展的主攻方向，注重在政策扶持和环境建设上下功夫，民营经济发展呈现出新的生机；坚持把扩大投资规模、优化投资结构作为拉动经济增长的关键举措，加强基础设施和重点项目建设，经济发展后劲不断增强；坚持把发展壮大市级经济作为政府工作的重要内容，强力推进宣城经济技术开发区建设，不断培育新的经济增长点，中心城市的辐射力和吸引力正在逐步增强；坚持以经济社会协调发展为目标，关心弱势群体，强化社会保障，重视就业和再就业工作，全市政治稳定，人心安定。这些成绩是省委省政府和市委的正确领导的结果，是全市人民团结奋斗、扎实工作的结果。在此，我谨代表市人民政府，向为宣城改革和发展付出辛勤劳动的广大工人、农民、知识分子、干部和离退休老同志，向给予市政府工作大力支持与有效监督的人大代表、政协委员和各民主党派、工商联、无党派人士，向为宣城各方面工作作出积极贡献的人民解放军指战员、武警官兵、政法干警和中央、省属各单位，向所有关心、支持宣城改革开放和现代化建设的同志们、朋友们，表示崇高的敬意和衷心的感谢！

在肯定成绩的同时，我们也清醒地看到，当前我市经济和社会发展中还存在不少困难和问题。主要表现在：产业结构层次较低，技术创新能力不强，经济持续快速增长的基础还不够稳固；城市建设与管理的任务比较艰巨，集聚和带动作用还不够明显；农村非农产业发展不快，农民收入增长缓慢；人才、土地、资金等生产要素供求矛盾还比较突出，交通等瓶颈制约因素尚未根本消除；就业与再就业的压力加大，部分群众生活比较困难；政府职能转变还不够到位，经济发展环境有待进一步优化等。对这些问题，我们将继续予以高度重视，在今后的工作中努力加以解决。

今年是实现“十五”计划的关键一年，也是我市全面建设小康社会起步阶段的重要一年，扎实做好以经济建设为中心的各项工作，意义十分重大。当前，我国经济已进入新一轮增长的上升期，以上海为龙头的长江三角洲地区着眼于率先基本实现现代化，产业升级和转移的步伐不断加快。经过多年的艰苦努力，特别是融入苏浙沪经济圈战略的实施，我市经济正呈现出加快发展的良好态势。我们必须牢牢把握战略机遇期，切实增强加快发展的紧迫感和责任感，在迎接挑战中抢抓新机遇，在扬长避短中增创新优势，在市场竞争中实现新跨越。

根据中央和省委省政府的部署，结合我市实际，今年政府工作的总体要求是：以邓小平理论和“三个代表”重要思想为指导，全面贯彻党的十六大、十六届三中全会和中央经济工作会议精神，树立全面、协调、可持续的发展观，围绕全面建设小康社会起步阶段的重点任务，进一步深化融入苏浙沪经济圈的发展战略，解放思想，抢抓机遇，坚持把推进工业化作为经济结构调整的核心任务，坚持把招商引资作为经济工作的第一抓手，坚持以深化改革为动力，坚持把加快城市化进程作为一项重要战略，统筹城乡发展，不断提高人民生活水平，努力实现经济快速协调健康发展和社会全面进步。 按照上述总体要求，今年全市经济和社会发展的主要预期目标是：国内生产总值增长9.5%，财政收入增长10%；全社会固定资产投资增长20%；外贸出口创汇1.1亿美元；城镇居民人均可支配收入增长8%左右，农民人均纯收入增长4%左右；城镇新增就业岗位1.7万个，登记失业率控制在4.3%以内，人口自然增长率控制在5‰以内。为实现上述目标，市政府将团结和依靠全市人民，着力抓好以下几个方面的工作：

一、坚持走新型工业化道路，促进产业结构优化升级

加速工业化是我市全面建设小康社会的首要任务。必须进一步强化工业意识，坚定不移地实施工业化战略。突出抓好宣城海螺水泥熟料生产线、海螺水泥四期、新鸿发120万套汽车注塑件、司尔特40万吨复合肥、飞鹰200万套车轮钢圈等20个续建和新开工的重点技术改造项目，推动产业升级和结构优化。立足现有工业基础，做大做强支柱产业和骨干企业，大力扶持汽车零部件及装备制造、医药化工、建材、农产品加工四大支柱产业加速发展，重点培育飞彩、古泉、新和成、中鼎、亚新科、红星宣纸、绩溪丝业、黄山胶囊、精方药业、广德菲达和三狮水泥、郎溪金润铜业等一批主业突出、核心竞争力强的骨干企业，鼓励联合重组，延伸产业链条，发展产业配套。高度重视中小企业发展，着力扶持一批充满生机与活力的中小企业群体，为我市工业发展奠定更加坚实的基础。

切实把宣城经济技术开发区建设摆到更加突出的战略位置，作为城市的一个新区来建设，继续加强水电路等基础设施和宣南工业园、飞彩科技工业园建设，营造规模优势，形成聚集效应。各县市区要借鉴苏浙地区发展块状经济的经验，因地制宜，集中力量抓好现有开发区和工业园区建设，构筑县域经济发展的新平台。开发区和工业园区要节约用地，预留好土地以供“大而好”的工业项目使用。积极探索开发区和工业园区建设的新模式，按照“一区多园”的体制和“谁引进、谁受益”的原则，鼓励引导不具备兴办工业园区的乡镇为开发区和工业园区引进项目，提高产业集中度。

提升服务业既是调整优化经济结构的重要内容，也是加速工业化的必然要求。要适应新一轮消费结构升级的新趋势，继续组织实施全市旅游发展总体规划，按照建设“两山一湖”东部旅游板块的要求，进一步加强重点景区景点的开发和建设，整合旅游资源，创新旅游品牌，加快与“长三角”旅游网络的对接。认真筹办好首届中国·宣城文房四宝之乡文化旅游节暨第三届宣城旅游招商促销洽谈会，积极参加省组织的中东部旅游交易会，大力开拓国内外旅游市场。强化政府对房地产一级市场的管理，全面放开搞活房地产二三级市场。继续抓好商贸流通、交通运输等传统服务业，大力发展电子商务、连锁经营、物流配送等新型流通方式。积极培育和规范会计、律师、咨询、经纪等各类中介服务组织。

二、全面发展农业和农村经济，努力增加农民收入

解决“三农”问题，核心是增加农民收入，关键是搞好结构调整，根本是建设现代农业。要认真贯彻落实《中共中央国务院关于促进农民增加收入若干政策的意见》，按照统筹城乡经济社会发展的要求，坚持“多予、少取、放活”的方针，继续推进农业和农村经济结构调整，不断开拓农业增效和农民增收的空间。适应当前农产品市场出现的新变化，优化品质、品种结构，提高粮食单产，推进商品粮基地建设，保护粮食综合生产能力。加快实施优势农产品区域布局调整，突出发展畜牧养殖、特经作物、经济林果和特种水产四大主导产业，积极扩大优质茶叶、烟叶、蚕桑、蔬菜和绿化苗木的种植面积，加快南漪湖综合开发和利用，着力打造基地化的特色农业。切实把保证农产品质量、安全作为调整农业结构的关键举措，加快农产品质量标准体系和检验检测体系建设，加强农产品质量安全认证，引导农民按标准化组织生产，大力发展无公害农产品、绿色食品和有机食品，提高农产品的质量和市场竞争力。坚持以工业的理念发展农业，积极推进农业产业化经营。重点扶持广德和威、宁国山里仁和太阳禽业、宣州华龙、郎溪昌发、旌德麻业等一批带动力强、覆盖面广的产业化龙头企业，主攻保鲜包装和精深加工等技术，促进产、加、销等各个环节的增值。进一步完善农业服务体系，加强农村经济信息网建设，大力发展农产品行业协会、农民专业合作组织和农民经纪人队伍，重点扶持营销大户和农村致富带头人，提高农民的组织化程度。切实把劳务输出作为增加农民收入的一项重要工作来抓，从信息引导、岗位培训、组织协调、劳动保障等各个环节，为农民外出务工经商提供有效服务，力争今年劳务必输出的人数和收入有较大幅度的增加。 高度重视畜禽疫病防治工作。最近一段时间，高致病性禽流感在国内外一些地区蔓延，我市已有疫情发生。要充分认识防治工作的长期性、艰巨性和复杂性，继续把高致病性禽流感防治工作列入重要日程，加强领导、密切配合、依靠科学、依法防治、群防群控、果断处置，以对人民群众高度负责的精神，抓紧完善防控预案，加强疫情监测，突出抓好大型养殖场和养殖大户的防疫工作，确保种禽安全，防止疫情发生。一旦发现疫情，要坚决扑杀，彻底消毒，严格隔离，强制免疫，确保疫情不扩散。始终把人民的生命健康放在第一位，进一步强化高危人群和疫区易感人群的医学检测，对进入市场的家禽及畜产品实行严格检验，坚决防止疫情对人的感染。高度关心疫区群众和禽业企业、养殖大户的切身利益，及时了解他们的情绪和困难，尽力帮助他们渡过难关。继续组织好非疫区家禽的销售工作，积极为禽业企业和养殖大户提供必要的资金支持。加快动物疫病防治体系建设，科学规划畜禽养殖业布局，推动畜禽养殖业持续稳定发展，努力把疫情损失降到最低程度。 继续稳定和完善农村土地承包关系，建立健全多种形式的土地流转机制，推动土地规模经营。实行最严格的耕地保护制度，切实保护基本农田，完善土地征用补偿办法，探索建立失地农民保障制度。深入推进农村税费改革和粮食补贴方式改革，取消农业税附加，进一步减轻农民负担。继续加大对农业和农村基础设施建设的投入，抓好乡村道路、人畜饮水和农村能源建设，推动农田水利基本建设向纵深发展。增强防灾抗灾意识，积极做好防汛抗旱工作。

三、强力推进招商引资工作，大力发展开放型经济

招商引资是我市经济和社会发展的重中之重。今年要在已有的基础上，升温加压，强力推进，确保全年引进外资4500万美元，引进省外资金30亿元。坚持把苏浙沪地区作为招商引资的主攻方向，突出工业招商，同时加大基础设施、农业、服务业和社会事业利用外资的力度。继续实行重大项目领导联系和协调会议制度，把项目签约率、资金到位率、项目开工率作为考核的主要内容，强化全过程跟踪服务。不断创新招商引资方式，做到传统招商与现代招商相结合，主动出击与委托招商、以商招商相结合，提高招商引资的实际效果。充分发挥企业特别是优势企业在招商引资中的主体作用，做到政府推动与企业招商、民间招商并重，大中小项目并举。今年要积极参加省组织的赴粤和赴港招商引资活动，力争取得好的成效。加强招商引资项目建设，规范项目资料，搞好项目论证，做深做细前期工作。锲而不舍地抓好优化投资环境工作，着力营造良好的政务环境、法制环境、市场环境和信用环境。在推进招商引资的过程中，要高度重视环境保护工作，严禁引进污染环境、破坏资源的项目。继续实施以质取胜和市场多元化战略，认真落实国家和省鼓励出口的各项优惠政策，大力扶持具有出口竞争能力的重点产品、重点行业和优势企业，积极扩大地产品出口，调整结构，开拓市场，努力保持外贸出口的良好势头。

四、进一步深化企业改革，加快发展民营经济

完善社会主义市场经济体制，是一项重大而紧迫的任务。必须立足宣城实际，进一步加大企业改革的力度，为经济和社会发展提供强有力的体制保证。继续以国有产权和职工身份“双置换”为主要内容，全面完成国有中小企业的改制任务。通过增资扩股、股权转让等多种形式，大力推进国有企业产权多元化，发展混合所有制经济，市属国有大中型企业要率先突破。坚持以明晰产权为重点，深入推进集体企业改革。已完成产权改革的企业，要完备和规范手续，解决好改制过程中的遗留问题。改革必须坚持规范操作、“阳光”操作，确保国有集体资产“流动不流失”，确保职工合法权益不受损害。加快完善国有资产管理体制，依法履行出资人职责，做到权力、义务和责任相统一，管资产和管人、管事相结合。

坚持把发展民营经济作为优化经济结构、促进经济增长的主攻方向。坚决取消限制民营经济发展的各项政策规定，确保民营企业在项目审批、土地使用、贷款、税收和对外贸易等方面,与其他企业享受同等待遇。进一步放宽市场准入，凡是对外资开放的领域，都要对民间资本开放，凡是法律法规未禁入的建设项目，一律对民间资本开放。改进民营企业的服务和监管，建立健全面向中小企业的服务体系，为民营企业及时提供资金、技术、信息和法律等方面的服务,积极扶持科技型、外向型、吸纳下岗人员就业型和农产品加工型企业的发展。鼓励有实力的民营企业参与国有集体企业改制和重组，努力培育一批成长性好、带动力强的大企业，造就一支高素质的民营企业家队伍。按照省政府的要求,今年起将把发展民营经济纳入对各级政府考核的指标体系,以促进民营经济更快更好地发展。

五、切实做好增收节支工作，努力增加金融信贷投放

财政工作要适应改革、发展和稳定的要求，进一步加大增收节支力度，确保财政收支平衡。大力培植和开拓财源，依法强化税收征管，严厉打击各种偷税、逃税和骗税行为，保持财政收入稳定增长。健全公共财政体制，继续完善综合财政预算、部门预算和国库集中支付制度，厉行节约，反对浪费，勤俭办一切事业。深化所得税分享改革，全面推行乡财县管新模式。推进财政资金使用绩效评估工作，加强对重点项目建设资金和其他各类专项资金的审计监督，提高资金使用效率。强化部门财务管理，重点加强对会议费、招待费、车辆购置费、办公楼装修费等支出项目的监督，建立健全科学的支出定额体系。继续加大机构、编制管理工作的力度，积极推进事业单位人事、分配制度改革，严格控制财政供给人员的增长。充分发挥金融对经济增长的促进作用，搞活信贷营销,增加信贷投入。依法维护金融债权，防范和化解金融风险。继续深化农村信用社改革，增强为农服务功能。积极创造条件，争取引入全国和区域性股份制商业银行在我市设立分支机构，不断扩充信贷资源。

六、加强城镇和基础设施建设，保持投资快速增长

按照皖东南中心城市和工贸旅游城市的目标定位，在城市总体规划的指导下，认真组织实施城市近期建设规划，加快编制各类专项规划和重要地段的控制性详规。抓紧做好昭亭北路改造和南北昭亭路节点段的施工设计工作，全线贯通南北昭亭路。全面完成敬亭山东大门、环城大道西南段和大坝塘游园建设工程，开工建设宣港路出口段西半幅、环城大道东南段、鳌峰东路延伸、日月东路和日月西路、敬亭北路、宣湖南路、绩溪路、府山头广场（体育场）和敬亭山西大门。启动实施市天然气利用工程、污水处理厂和垃圾处理厂、三水厂等项目建设。树立经营城市的理念，坚持走开发建设市场化、资金筹措多元化、资源利用商品化的新路子。进一步加强城市管理，逐步实行政企、政事和管养分开，推进市政设施维护、环卫保洁的市场化改革。继续抓好各县市城区及中心镇的规划与建设，加速推进城镇化进程。

继续加强交通、水利、电力、通信等基础设施建设。全面推进交通“九路八桥”工程，确保318国道广祠段高速公路、214省道望十线、215省道广宁线和宁国排湾店至绩溪38号桥段改造、宣州东门渡大桥接线和东溪河复桥、西津河大桥年内建成通车。继续抓好县乡公路“通达工程”，开工建设钟梅路、宣水路和石千路。积极做好全市拟建高速公路的规划和设计，抓紧完成报批立项工作，争取早日开工建设。加强宣城市区城市防洪体系建设，抓紧抓好郎溪县城区防洪工程等一批在建重点项目的实施。抓住当前国家电力紧缺和我省加快建设能源基地的机遇，开工建设宣城500千伏变电所及配套输送工程，扎实推进宣城电厂的前期准备工作。切实做好宣杭铁路复线工程建设的协调服务工作，保证工期需要。继续推进通信光缆传输网、数据接入网和宽带城域网建设，加快国民经济和社会信息化进程。

七、大力推进社区建设，强化城市服务和管理功能

全面推进社区建设是加快城市发展、强化城市管理、维护社会稳定的重要保证。切实把社区建设纳入城市和城区工作的总体规划，着力解决“有人办事、有钱办事、有地方办事、有章理事”等问题，逐步形成党委政府领导、民政部门牵头、有关部门配合、社会力量支持、群众广泛参与的工作格局。按照“两级政府、三级管理”的框架，加快建立适应市场经济要求的社区组织体系、管理体系和服务体系，促进城市管理重心下移，构筑城市基层工作的新平台。充分发挥社区在实现下岗失业人员再就业、落实城市“低保”工作中的重要作用，积极开发社区就业岗位，千方百计搞好就业服务，切实抓好社区面向社会特殊群体的社会救助、社会福利服务和各种便民利民服务。明确界定不同性质社区组织的职能，进一步理顺和规范社区居委会与街道办事处、物业管理公司的关系，深入开展社区共建共管活动。大力发展城市基层民主，推进社区居民自治，做好社区治安和稳定工作。今年宣州区和宁国市的社区建设要全面推开，其他县可结合自身实际，在城区选择2—3个社区进行试点，积累经验，循序渐进，推动社区建设健康发展。

八、统筹经济社会协调发展，全面推进各项社会事业

加强公民道德建设和诚信教育，继续深入开展文明城市、文明行业、文明单位和文明村镇等群众性精神文明创建活动，倡导健康文明的生活方式。大力推进科技进步与创新，召开市首届科技进步大会，激发全社会的科技进步意识。全面实施教育六项工程，提升教育质量和水平。高度重视义务教育成果的巩固和发展，继续完善农村义务教育管理体制，建立和完善农村教育投入的保障机制。进一步整合教育资源，继续抓好中小学布局调整和危房改造二期工程。大力发展幼儿教育和高中阶段教育，抓好职业教育和成人教育，鼓励发展民办教育，着手兴建宣城职业技术学院新校区，加快发展高等教育。牢固树立“人才是第一资源”的观念，高度重视人才的培养、使用和引进工作，努力造就一支适应新时期发展需要的高素质人才队伍。推进文化基础设施建设，积极筹办好第十一届中国曹禺戏剧奖“飞彩杯”全国小品小戏大赛和第二届宣城艺术节。保护和挖掘地方传统文化，加强皖南花鼓戏的人才培养和市花鼓戏剧团建设。加快发展广播电视事业，广泛开展全民健身活动，积极筹建市广电中心和体育中心。深化卫生体制改革，加强公共卫生服务体系、疾病预防控制体系和农村合作医疗体系建设，提高城乡公共卫生保障水平。加强重大疾病预防控制，开工并完成市疾病控制中心建设。继续做好非典防治工作，严格落实各项防控措施，确保人民群众身体健康和生命安全。

继续推进人口与计划生育综合改革，大力加强计划生育基层队伍和信息网络建设，稳定低生育水平。依法保护与合理开发自然资源，抓紧编制全市生态建设总体规划，扎实推进森林生态网络和生态示范区建设，着力打造“生态宣城”。全面贯彻落实党的民族、宗教、外事、侨务和对台政策，继续做好档案、地方志、文物保护和防震减灾等工作。认真搞好全国第一次经济普查。积极支持工会、共青团、妇联等群众团体的工作，关心支持未成年人、老年人和残疾人事业，维护他们的合法权益。坚持国防建设与经济建设协调发展的方针，加强国防教育，支持国防建设，做好民兵预备役和“双拥”工作，巩固和发展军政军民团结。

九、着力解决群众关心的突出问题，维护社会政治稳定

认真落实扩大就业、促进再就业的各项政策，严格实行就业和再就业目标责任制，保证税费减免、小额贷款、岗位补贴等扶持政策落实到位。统筹经济发展和增加就业，促进个体私营经济、劳动密集型产业和中小企业吸纳就业，不断增加就业容量。完善就业服务体系，加强技能培训、职业介绍、就业指导等相结合的综合服务，提高择业者就业和创业能力。加大再就业援助力度，大力开发公益性岗位，积极帮助困难群体实现再就业。继续做好大中专毕业生和城镇新增劳动力的就业工作。规范企业用工行为，保障劳动者合法权益。

进一步扩大企业职工基本养老保险覆盖面，建立并实施企业基本养老保险市级调剂机制。健全失业保险制度，实现国有企业下岗职工基本生活保障向失业保险并轨，着力抓好个体私营企业参保工作。继续完善城镇职工基本医疗保险制度改革，采取大病统筹和社会救助等办法，妥善解决困难企业职工和困难群体医疗保障问题。完善城市居民最低生活保障制度，合理确定保障标准，努力做到动态管理下的应保尽保。强化社会保险基金征缴和管理，严禁挤占挪用，确保基金安全。启动实施工伤保险，继续推进企业退休人员社会化管理服务工作。切实做好扶贫开发工作，加快建立城乡特困群体救助制度。

严格落实安全生产责任制和责任追究制，强化安全生产监管机制，深入开展交通、煤矿安全等专项整治，坚决防止重大事故发生。高度重视人民群众来信来访工作，妥善处理改革与发展中出现的各种矛盾和问题，及时疏导和化解社会不稳定因素。扎实推进“四五”法制宣传教育，广泛开展评选“遵纪守法好公民”活动，提高全民的法制观念。加强社会治安防控体系建设，继续抓好基层安全创建工作，依法打击各种犯罪活动，确保社会政治稳定。

十、加快转变政府职能，不断提高行政效率和服务水平

加快完善社会主义市场经济体制，对政府自身建设提出了新的更高要求。我们将致力于加快政府职能转变，大力推进管理创新，降低行政成本，提高行政效能，努力形成行为规范、运转协调、公正透明、廉洁高效的行政管理体制。 始终把人民的利益作为政府工作的出发点和落脚点，永葆人民公仆本色。对企业改制、城镇改造拆迁、农村公益事业建设等关系到群众利益的问题，一定要充分听取群众的意见和建议，把工作做深做细。继续坚持领导联系基层、群众来信来访接待日和市长热线电话等制度，大力推广和规范各种类型的社会服务活动。树立正确的权力观、地位观和利益观，真正做到权为民所用，情为民所系，利为民所谋。 认真贯彻实施《行政许可法》，继续抓好学习、宣传和培训工作，对行政审批项目、行政许可规定和实施机关进行全面清理，建立健全实施行政许可的运行、监督机制，依法规范行政行为。进一步扩大政务公开范围，完善重大事项社会公示和听证制度，增强政府工作透明度和社会公众参与度。继续推进行政审批制度改革，加强政务服务中心建设,大力推行限时审批、并联审批和限期办结、责任追究等制度,提高工作和办事效率。各级政府要自觉接受人大监督，积极支持人民政协履行政治协商、民主监督、参政议政的职能，认真办理人民代表议案、建议和政协委员提案，主动听取各民主党派、工商联、无党派人士及各人民团体的意见和建议，欢迎和支持人民群众、新闻媒体对政府工作进行监督。 坚持解放思想、实事求是、与时俱进，注意把握新趋势，善于总结新经验，不断提高工作的预见性和创造性，提高议事和决策水平，以创新的思维谋划工作，用发展的办法解决问题。树立正确的政绩观，坚决克服官僚主义和形式主义，做到不切实际的口号不提，力所不及的事情不为，急功近利的事情不做。切实减少会议、文件和不必要的检查、评比活动，集中精力研究和解决实际问题。

全面落实党风廉政建设责任制，严格执行中纪委第三次全体会议提出的四大纪律八项要求，突出抓好各级干部特别是领导干部的廉洁自律，坚决查处违纪违法案件，重点纠正部门和行业不正之风。完善“收支两条线”管理、建设工程项目公开招投标、经营性土地使用权公开出让、产权交易和政府采购等制度，进一步加强审计监督。建立健全教育、制度、监督并重的惩治和预防腐败的工作体系，切实规范各级公务员的从政行为，努力树立为民、务实、清廉的良好形象。

各位代表，宣城这一方热土，正焕发着前所未有的勃勃生机。在全面建设小康社会的征程中，机遇与挑战并存，困难与希望同在。让我们高举邓小平理论伟大旗帜，努力实践“三个代表”重要思想，紧密团结在以胡锦涛同志为总书记的党中央周围，在省委省政府和市委的坚强领导下,紧紧团结和依靠全市人民,励精图治，锐意进取，开拓创新，扎实工作，为全面建设小康社会、创造宣城更加美好的未来作出新的更大贡献！
